# Comparison between dexmetomidine and midazolam on early extubation and hemodynamic profile. a randomized double-blind study

**DOI:** 10.1186/2197-425X-3-S1-A327

**Published:** 2015-10-01

**Authors:** M Mavri, K Rellos, I Pantazopoulos, C Kampolis, I Floros, N Iacovidou, T Xanthos, C Pantazopoulos

**Affiliations:** Central Clinic of Athens, Research and Treatment Medical Center, Anaesthesiology and Intensive Care, Athens, Greece; Evagelismos General Hospital of Athens, Intensive Care Unit, Athens, Greece; Laiko General Hospital of Athens, University of Athens, Pathophysiology Clinic, Athens, Greece; Laiko General Hospital of Athens, University of Athens, Intensive Care Unit, Athens, Greece; University of Athens, Medical School, Neonatology, Athens Greece; University of Athens, Medical School, MSc Cardiopulmonary Resuscitation, Athens, Greece

## Introduction

Patients under mechanical ventilation in the Intensive Care Unit (ICU) require sedation in order to facilitate ventilator and endotracheal tube tolerance. The sedative agent that must be used should not interfere with early extubation of patients. Midazolam is a benzodiazepine, a common sedative agent used in the ICU, while dexmetomidine is an alpha 2 adrenergic receptor agonist, that is used over the last years. It has both sedative and analgesic effects.

## Objectives

The scientific objective of the study was to compare dexmedetomidine and midazolam regarding their extubation profile, as well as their cardiovascular response.

## Methods

The present study included forty (40) mechanically ventilated patients of both sexes, aged 20-60 years, who were meeting the standard criteria for weaning. Patients were randomly allocated into 2 groups. Each group included 20 patients (n = 20). Patients in group D (Dexmetomidine) received intravenous infusion of dexmedetomidine (0.2-0.7 mcg/kg/h), while in group M (Midazolam), patients received midazolam (0.04-0.2 mg/kg/h) in order to achieve a Ramsay sedation scale of 2-4. Extubation was performed after standard extubation protocol was completed. Time for extubation and vital parameters were recorded periodically.

## Results

Time to extubation in the dexmedetomidine group was significantly lower than in the midazolam group. Blood pressure and heart rate was most of the times significantly lower in the dexmedetomidine group than in the midazolam group. (p < 0.05)

## Conclusions

Dexmedetomidine has obvious clinical benefits compared to midazolam regarding extubation. This occurs due to its shorter extubation time, better hemodynamics, easy arousability and lack of respiratory depression.
